# Separating microparticles by material and size using dielectrophoretic chromatography with frequency modulation

**DOI:** 10.1038/s41598-021-95404-w

**Published:** 2021-08-19

**Authors:** Jasper Giesler, Laura Weirauch, Jorg Thöming, Michael Baune, Georg R. Pesch

**Affiliations:** 1 Chemical Process Engineering, Faculty of Production Engineering, University of Bremen, Leobener Straße 6, 28359 Bremen, Germany; 2 MAPEX Center for Materials and Processes, University of Bremen, PO box 330 440, 28334 Bremen, Germany

**Keywords:** Chemical engineering, Mechanical engineering

## Abstract

Separation of (biological) particles ($\ll {10}~{\upmu }\text {m}$) according to size or other properties is an ongoing challenge in a variety of technical relevant fields. Dielectrophoresis is one method to separate particles according to a diversity of properties, and within the last decades a pool of dielectrophoretic separation techniques has been developed. However, many of them either suffer selectivity or throughput. We use simulation and experiments to investigate retention mechanisms in a novel DEP scheme, namely, frequency-modulated DEP. Results from experiments and simulation show a good agreement for the separation of binary PS particles mixtures with respect to size and more importantly, for the challenging task of separating equally sized microparticles according to surface functionalization alone. The separation with respect to size was performed using 2 ${\upmu }$m and 3 ${\upmu }$m sized particles, whereas separation with respect to surface functionalization was performed with 2 ${\upmu }$m particles. The results from this study can be used to solve challenging separation tasks, for example to separate particles with distributed properties.

## Introduction

Separation of particles from each other is important in a wide variety of areas. For example, it is required in electronic waste recycling to recover valuable metals^1–3^, to enrich desired minerals in the mining sector^4,5^, to detect circulating cancer cells^6^, in waste water treatment^5^, and many other fields. For large particles ($\gg$ 10 ${\upmu }$m), inertia- or gravity-driven processes are one option to achieve a classification with respect to density or particle size. Since both, gravity and inertia scale with particle mass, their influence decreases with decreasing particle size and becomes negligible when particles reach nanometre scale^7^. In this range, other forces (e.g. electrostatic, van-der-Waals interaction or Brownian motion) can dominate the particle behaviour. Thus, to separate micro or sub-micron particles, other approaches become attractive. We like to note that many biological separation tasks^8–11^ or valuable dust fractions^2^ are within this size range. For such particle sizes, (gel-)electrophoresis^12,13^, field-flow-fractionation (FFF)^14^, or size-exclusion chromatography^15^ are some common methods. Dielectrophoresis (DEP) is a versatile technique that is not only capable of addressing micro and sub-micron particles^16,17^, it also offers the potential to be scaled up^18^. Further, DEP can be used to manipulate both biological^9,19,20^ and non-biological particles^21,22^.

DEP describes the movement that rises when a suspended polarizable particle is placed into an inhomogeneous electric field. The dielectrophoretic force $\mathbf{F }_{\mathrm{DEP}}$ acting on a spherical particle is commonly approximated as^16^

1 $$\begin{aligned} \mathbf{F }_{\mathrm{DEP}}=2\pi r^3_{\mathrm{p}} \varepsilon _{\mathrm{m}} {\mathrm {Re}}(CM) \nabla \mathrm {|\mathbf{E} _{rms}|}^2 , \end{aligned}$$

with $r_{\mathrm{p}}$, the particle radius, the vector of the electric field $\mathbf{E} _\text{rms}$ and the permittivity of the surrounding medium $\varepsilon _{\mathrm{m}}$. ${\mathrm {Re}}(CM)$ is the real part of the so-called Clausius–Mossotti factor, which incorporates the frequency-dependent polarization of the particle and the medium. Using the complex permittivity $\tilde{\varepsilon }$ it can be calculated for homogeneous spherical particles as

2 $${\text{Re}}(CM) = {\text{Re}}\left( {\frac{{\tilde{\varepsilon }_{\mathrm{p}} - \tilde{\varepsilon }_{\mathrm{m}} }}{{\tilde{\varepsilon }_{\mathrm{p}} + 2\tilde{\varepsilon }_{\mathrm{m}} }}} \right),$$

with $\tilde{\varepsilon }=\varepsilon _0\varepsilon _{\mathrm{r}}-i\frac{\sigma }{\omega }$, where $\sigma$ is the conductivity, $\varepsilon _0$ the vacuum permittivity and $\omega =2\pi f$ represents the angular frequency of the applied electric field. This factor ranges between - 0.5 and 1 and determines the movement direction of the particle: When ${\mathrm {Re}}(CM)>0$, particles experience positive dielectrophoresis (pDEP) and move towards local field maxima, when ${\mathrm {Re}}(CM)<0$, particles experience negative dielectrophoresis (nDEP) and are repelled from field maxima. The frequency where ${\mathrm {Re}}(CM)$ equals zero is called crossover frequency. At this frequency, the particles do not experience a dielectrophoretic force. Due to its dependency on field frequency and medium properties, ${\mathrm {Re}}(CM)$ can change its value or sign during an experiment, which can result in a movement direction change of target particles. The net conductivity of a microparticle of non-conducting bulk material in an electrolyte suspension can be calculated as^16,23^

3 $$\begin{aligned} \sigma _{\mathrm{p}}=\sigma _{\mathrm{bulk}}+\frac{2K_{\mathrm{s}}}{r_{\mathrm{p}}}. \end{aligned}$$

The conductivity is composed of the bulk material conductivity, $\sigma _{\mathrm{bulk}}$, and a part caused by the intrinsic double layer that forms around suspended particles. The surface conductance $K_{\mathrm{s}}$ comes from the ions in the electric double layer of the particle and can increase the overall conductivity^16^. As a consequence, even particles with negligible bulk conductivity, such as the polystyrene (PS) particles used in this work, can show pDEP.

Equations (1), (2) and (3) show that the dielectrophoretic motion depends on material (e.g. conductivity and permittivity), process parameters (e.g. medium conductivity, field strength and frequency) and size. The diversity of influencing variables provides the opportunity to address different separation tasks. Depending on the process design, even specific multidimensional tasks could solved in one set-up. Simultaneously, DEP-based separation requires careful design to enable a functioning separation processes. In its 50 years of existence, many different techniques and designs have been established to perform a dielectrophoretic separation of particles. One way to categorize the existing DEP techniques is whether a continuous or a chromatographic separation is performed. Whereas continuous separation methods often focus on spatial separation or selective trapping^17,24–26^, chromatographic methods are usually batch or semi-batch processes and result in particle type-dependent residence times in a separator. They are a promising approach to achieving separation of high purity or adjustability^27,28^. In this work, we use experiments and simulation to gain further insight into the retention mechanisms of a chromatographic separation based on a frequency-modulation method.

Dielectrophoretic particle chromatography (DPC) was introduced by Washizu et al.^9^ in 1992 and has been used since^8,27–31^. A prominent example is the isolation of tumor cells from blood by Shim et al.^19^. DPC exploits different polarizabilities of target particles for separation. For example, a specific particle type shows pDEP ($\text{Re}(CM)>0$) and gets reversibly trapped in the separation column, whereas other particles show no DEP or nDEP and are consequently eluted from the column. By changing the frequency, the formerly trapped particles in the channel can be levitated by nDEP, resulting in their subsequent elution. Some approaches vary the frequency as a function time, to separate different cell types from one another^8,19^ or achieve a separation with respect to size^32,33^. As Yang et al.^28^ also pointed out, sweeping the frequency can be used to compensate distributed cell properties and consequently achieve more homogeneous retention times in dielectrophoretic field-flow fractionation (FFF) by also reducing particle adhesion at field maxima at the same time. The above mentioned studies show the potential of varying the frequency in DPC. Additionally, Aldaeus et al.^27^ numerically showed the benefit of multiple trap-and-release cycles in DPC. In a previous publication, we demonstrated the capabilities of a design that combined multiple trap-and-release cycles with the advantage of changing the frequency^33^. The chromatographic separation allows to address particle mixtures with only small dielectric differences or distributed particle properties. Additionally, since all particles elute from the same outlet, the design of the device is simple and easy to scale.

**Figure 1:**
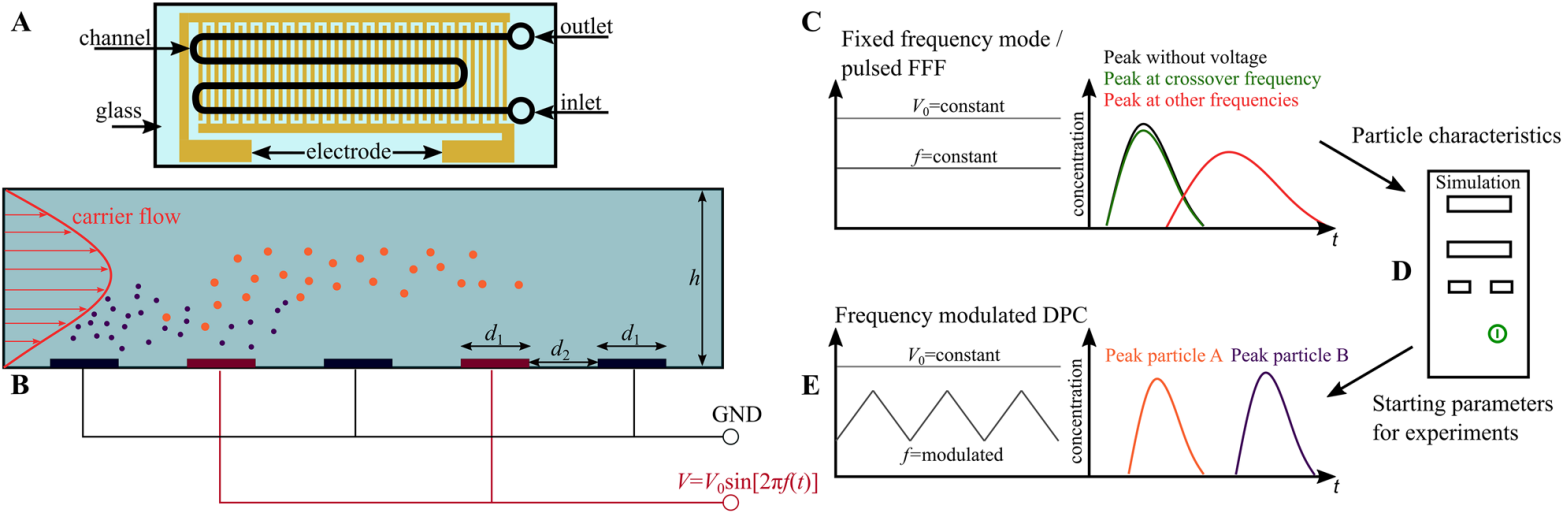
(**A**) Top view of the microfluidic device (sketch). (**B**) The microfluidic separation column (side view, height $h= {80}~{\upmu }{\text m}$ and electrode width/spacing $d_1 = d_2= {100}~{\upmu }{\text m}$) is continuously flushed with a carrier fluid. Once per experiment a particle suspension is injected. The device is used for two different types of experiment. (I) The crossover frequency of particles is determined using field-flow fractionation (FFF) at a fixed frequency *f* by comparing the elution profiles with and without applied voltage ($V_0$) (**C**). The obtained particle characteristics where used as input parameters for a full-scale simulation model realized in COMSOL Multiphysics to find suitable process parameters (**D**). (II) Eventually, the set of process parameters is used as starting point for experiments to achieve a chromatographic separation by using frequency-modulated ($f=f(t)$) dielectrophoretic particle chromatography (DPC) (**E**).

The functionality of such an approach is explained in detail in the “Functionality of frequency-modulated DPC” section. Briefly, a number of particles is injected once into a flow chamber and transported across an interdigitated electrode array by a carrier flow with a laminar flow profile (Fig. 1A,B). The electrode array generates an inhomogeneous electric field and thus a DEP force on the particles. The frequency of the applied field is continuously modulated between two values. For each particle in an arbitrary particle mixture, three general responses are possible: particles experience mainly pDEP in the modulation spectrum and are drawn towards the field maxima found at the electrode edges. Due to their interaction with the field maxima and the low fluid velocity close to the walls of the channel, they experience a retardation in the channel (as discussed below) and elute later than they would without field. The difference in elution time depends on the applied voltage and frequency range. Particles that, in contrast, experience mainly nDEP in the modulation spectrum interact with the field minima found at the channel ceiling and also elute later. Particles that experience a balanced pDEP/nDEP response in the modulation spectrum are neither drawn to the ceiling nor bottom of the channel and thus experience almost no retardation. They elute almost at the same time as they would without applied field. Thus, this technique allows to separate a particle mixture that is injected intermittently (or once) at the inlet (Fig. 1E); as long as the different particles show differences in their crossover frequency.

In our previous publication^33^, we demonstrated how the approach can be used to separate polystyrene microparticles based on size. Both design and drawn conclusions were based mostly on observations without detailed numerical calculations of the underlying physics. In this study, we will simulate the size-selective separation of polystyrene particles and verify the simulation results against experiments. We will further capitalize on the simulation to predict parameters for separating polystyrene particles of equal size based only on their surface conductance. Finally, we will compare the simulated and experimental results of this separation. According to Eq. (3), the conductivity and thus crossover frequency of polystyrene particles depends on their size and the yet unknown surface conductance $K_{\mathrm{s}}$. Thus, to perform a simulation we need to determine the crossover frequency and the particle’s $K_{\mathrm{s}}$-value. To do this, we use a fixed-frequency method (Fig. 1C), which is explained in detail in the “Determination of crossover frequency” section: Here, the frequency is kept constant per particle injection (but is changed between experiments) and the particle residence time is observed as a function of applied frequency. When the applied voltage is chosen carefully, particles will either be retarded by positive or negative DEP, or, in case the applied frequency closely matches the crossover frequency, particles will not be retarded. Thus, by comparing the elution time as a function of frequency and comparing it against the elution time without superimposed electric field, it is straight-forward to determine the crossover frequency. The determined $K_{\mathrm{s}}$-value can be adjusted slightly to improve the match between experiments and simulation. To summarize our approach:

i. Find the crossover frequency and $K_{\mathrm{s}}$ of the particles by performing fixed-frequency experiments (Fig. 1C).
ii. Use the obtained $K_{\mathrm{s}}$-value to determine suitable frequency ranges and perform frequency-modulated DPC experiments to separate particles by size (Fig. 1E).
iii. Simulate the particles movement and compare the elution profiles of the experiment and the simulation. Apply moderate changes to the simulation (e.g. simulated particle polarizability) to increase match with experiment (Fig. 1D).
iv. Use simulation to design a different separation task:
  - Find crossover frequency of polystyrene particles with different surface functionalization but same size.
  - Input crossover frequency into the simulation to find suitable center frequencies for separation in the experiment.
  - Perform the separation experimentally with optimized parameters from iii.b.

## Results and discussion

We first determine the surface conductance for the size-selective separation using fixed-frequency experiments (Fig. 1C). Based on these results, we perform DPC experiments using the frequency-modulation technique (Fig. 1E). We will then perform simulations, using the same process parameters, to see how the simulation matches the experiments. Finally, we use the simulation to find process parameters to separate a binary mixture of particles according to their surface modification.

**Figure 2:**
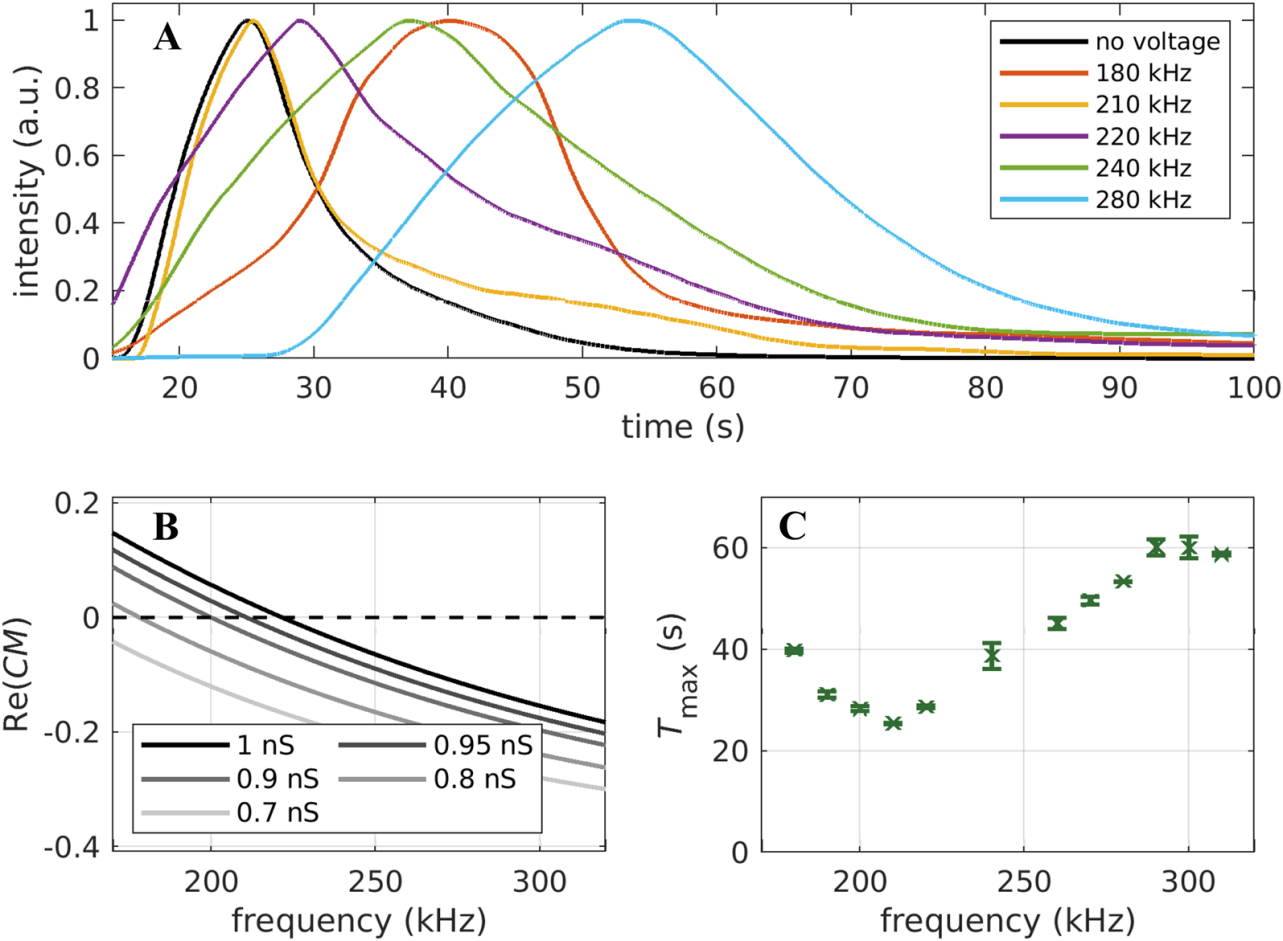
(**A**) Residence time distributions at 120 V$_{\mathrm{pp}}$ for different applied frequencies in fixed-frequency dielectrophoretic particle chromatography. (**B**) Calculated values of the real part of the Clausius–Mossotti equation for different surface conductances ($d_{\mathrm{p}}= {3.1}\;{\upmu }\text {m}$ PS particles without surface functionalization in a 2 ${\upmu }$S cm$^{-1}$ suspension). The dashed line represents ${\mathrm {Re}}(CM)=0$. (**C**) Maximum of the residence time distributions at 120 V$_{\mathrm{pp}}$ for all measured frequencies (extracted from the elution profiles). Experiments were repeated 3 times.

### Size-selective separation

Two monodisperse PS particle suspensions with diameters of 3.1 ${\upmu }$m and 2.12 ${\upmu }$m without an additional surface functionalization were selected for generating experimental data to compare with the simulation. Since the surface conductance is an important yet unknown characteristic of the particles, it was measured using fixed-frequency field-flow fractionation (see “Determination of crossover frequency” section). Choosing the right voltage for the experiments is important, as a too high voltage would cause immobilization and too low voltage would result in only slightly differences in the residence time distribution. For the larger particles a voltage of 120 V$_{\mathrm{pp}}$ was selected. The smaller particles required a higher voltage of 160V$_{\mathrm{pp}}$, since the DEP force scales with particle volume. Frequencies between 180 and 310 kHz were tested. Figure 2 shows the concentration profiles at the outlet for 3.1 ${\upmu }$m particles at different frequencies. At 210 kHz, the residence time is minimal and the concentration profile almost matches the profile without any applied voltage, indicating that the cross-over frequency is close to 210 kHz. At both, lower and higher frequencies, the concentration profiles are shifted towards longer times (i.e., particles elute later and are retarded to either nDEP in case of higher frequencies, or pDEP in case of lower frequencies). Combining the results with the real part of the Clausius–Mossotti factor (Eqs. (2) and (3) and Fig. 2), we calculate a surface conductance of $K_{\mathrm{s}}={0.95}~\text {nS}$, which is in good accordance with the literature value of 1 nS^16,23,34^. The results for the 2.12 ${\upmu }$m particles (Supplementary Fig. 2) show a minimum in residence time around 290 kHz. Since the dielectrophoretic mobility for smaller particles is lower, the crossover is less clear. Nevertheless, knowing the crossover is close to this value, a surface conductance of 0.9 nS was assumed for further steps.

**Table 1:** Anticipated particle behaviour in the DPC experiments based on their crossover frequency. The 2.12 ${\upmu }$m particles show their crossover at 290 kHz and the 3.1 ${\upmu }$m particles at 210 kHz.

Center frequency	2.12 ${\upmu }$m particles	3.1 ${\upmu }$m particles
210 kHz	pDEP dominated	Balanced
245 kHz	pDEP dominated	nDEP dominated
280 kHz	Balanced	nDEP dominated

Now that we know $K_{\mathrm{s}}$ and crossover frequency of both particles, we can select suitable frequency ranges for separation. Three frequency ranges were chosen. Two center frequencies $f_{\mathrm{c}}$ of the modulation spectrum ($f_{\mathrm{c}} = {210}~\text {kHz}$ and $f_{\mathrm{c}} = {280}~\text {kHz}$) were selected because these frequencies are close to the respective crossover of the two particles. A third frequency was chosen in between (245 kHz). The bandwidth of 240 kHz in combination with a modulation frequency of 300 mHz were kept constant, because we know from previous experiments that these parameters allow a separation^33^. Both parameters are constant for all conducted experiments within this work. The selection of frequency windows allows to test the three predicted behaviours of the particle in the channel (see “Functionality of frequency-modulated DPC” section and Table 1). The spectrum centred at 210 kHz should produce no or only small retardation for the larger 3.1 ${\upmu }$m particles, since they experience a balanced pDEP and nDEP force. In contrast to this, the movement of the 2.12 ${\upmu }$m particles should be dominated by pDEP, resulting in a retardation. At 280 kHz, we expect an increase in residence time for the larger 3.1 ${\upmu }$m particles as they are retarded due to an nDEP dominated response. The smaller 2.12 ${\upmu }$m particles experience balanced DEP and show no retardation. In the third spectrum (center frequency 245 kHz), which produces pDEP for the small and nDEP for the bigger particles, only poor separation is expected, as now both particles are retarded.

**Figure 3:**
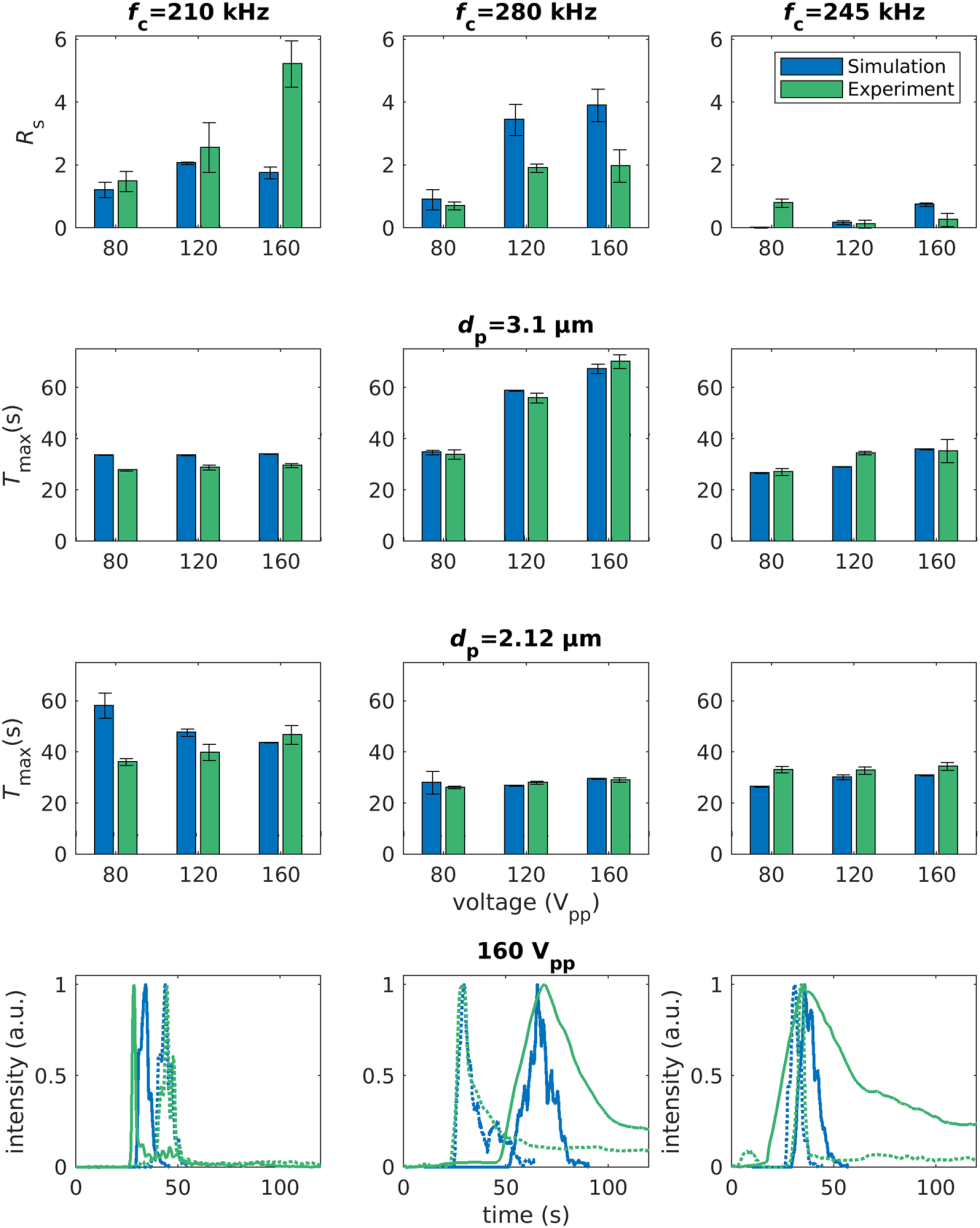
Top: Resolution and standard deviation of experimental and simulated frequency-modulated chromatography experiments at 80, 120 and 160 V$_{\mathrm{pp}}$ for three different modulation spectra. Surface conductance of the 2.12 ${\upmu }$m and 3.1 ${\upmu }$m particles are simulated with 0.775 nS and 1 nS, respectively. Middle two rows: Corresponding maxima of the residence time distributions and standard deviations for 3.1 ${\upmu }$m and 2.12 ${\upmu }$m particles. Bottom: Residence time distributions of experiment (green) and simulation (blue) for 3.1 ${\upmu }$m (solid line) and 2.12 ${\upmu }$m (dashed line) particles at different centre frequencies (210, 280 & 245 kHz) and 160V$_{\mathrm{pp}}$. Simulations and experiments were repeated 5 times to check for statistical validity.

Figure 3 shows experimental and simulated chromatography results of the size-selective separation of 2.12 ${\upmu }$m and 3.1 ${\upmu }$m particles. As an example, the bottom row shows both experimental and simulated elution profiles at 160 V$_{\mathrm{pp}}$ and at the three different centre frequencies. From these profiles, we can extract the separation resolution $R_{\mathrm{s}}$ (see “Experimental details” section for a definition of $R_{\mathrm{s}}$ and top row for the results) as well as the maxima in the respective peaks (middle rows). For the experiments, the best resolution at all voltages is achieved at $f_{\mathrm{c}} = {210}~\text {kHz}$ (top left). In this setting, the larger particles experience balanced pDEP and nDEP, thus almost no retardation, and are consequently eluted only about two seconds later than without an applied voltage (without applied voltage $T_{\mathrm{max}}={26.91}~\text {s}\pm {0.28}~\text {s}$). The 2.12 ${\upmu }$m particles, which experience more pDEP than nDEP, instead show a significant retardation. With increasing voltage, $T_{\mathrm{max}}$ of the 2.12 ${\upmu }$m particles and thus the experimentally determined $R_{\mathrm{s}}$ increase. In contrast, at $f_{\mathrm{c}} = {280}~\text {kHz}$ (middle panel), the 2.12 ${\upmu }$m particles now experience balanced pDEP and nDEP behaviour and are eluted much earlier than the larger particles, which now experience an nDEP dominated movement. The $T_{\mathrm{max}}$ of the 3.1 ${\upmu }$m particles and thus $R_{\mathrm{s}}$ here also increase with voltage, but the resolution is generally lower compared to $f_{\mathrm{c}} = {210}~\text {kHz}$. At $f_{\mathrm{c}} = {245}~\text {kHz}$, as expected, we observe only small retardation for both particle types, which consequently leads to a low experimental resolution among all voltages. These experiments show the three different types of particle movement in frequency modulated DPC. The set of experiments validates the theory stated before and is used for comparing chromatograms of experiment and simulation.

To achieve a good agreement in the residence time distributions between the experiments and our simulations, we applied empirical corrections to the surface conductance (see below). Especially maxima of the residence time distributions ($T_{\mathrm{max}}$) agree quite well across all frequencies and voltages. For the 3.1 ${\upmu }$m particles we applied an offset of 0.05 nS to the surface conductance (experimentally determined $K_{\mathrm{s}}= {0.95}~\text{nS}$ vs. simulated $K_{\mathrm{s}}= {1}~\text{nS}$), which is within the uncertainty of the method to determine the surface conductance. To the smaller particles we applied an offset of −0.125 nS , resulting in a simulated $K_{\mathrm{s}}$-value of 0.775 nS versus 0.9 nS found in the experiment. Since this would correspond with a crossover frequency of about 250 kHz, this can not be explained with the uncertainty of the surface conductance alone. Lowering the surface conductance in our simulation equals reducing the time the smaller particles spend adhered to the wall, resulting in a faster elution of the particles, which was observed experimentally. This is because they show predominately pDEP and by lowering the simulated conductivity of the particles, they show nDEP for longer periods of time. As the fixed-frequency experiments already suggest, not all particles adhere to the wall at fixed frequency, even when a frequency different from the crossover frequency is applied. Transferring this observation to the simulation, this means that the trapping of the particles at the wall is not as strong as predicted by the simulation. This is shown here for 2.1 ${\upmu }$m particles (Fig. 3). We also tested this for 6.14 ${\upmu }$m particles (see supplement). In general, corrections to the surface conductance are required so that they reduce the residence time in simulation. This means, particles that are predominantly experiencing pDEP require a correction so that the surface conductance is lowered. Particles that are experiencing more nDEP than pDEP require a correction that raises the surface conductance so that they experience nDEP for a shorter duration and thus spend less time adhered to the wall in the simulation. One explanation is that particles hop from one trapping location to another. (Electro-)thermal fluid movement and unspecific adhesion or hydrodynamic lift might be the reason behind this behaviour. Additionally, Adams et al.^35^ showed, that the sweeping rate (frequency change per time) can affect the polarization of microspheres significantly, which may contribute to the observed effects. To account for the experimentally observed behaviour in the simulation, we chose to adjust the surface conductance of the particles to reduce the time particles spend adhering to the wall. The data of the size-selective experiments show that the particle-wall interactions should be studied in more detail in the future to remove the surface conductance of the particles as a fitting parameter from the simulation. By now, in addition to the experiments that we require to determine the surface conductance, we also require experiments providing retention behaviour of the particles during frequency modulation to calibrate the surface condutance offset. Based on this calibration experiments we perform extensive parametric studies.

Interestingly, at $f_{\mathrm{c}} = {210}~\text {kHz}$, the simulated $T_{\mathrm{max}}$ of the 2.12 ${\upmu }$m particles decreases with voltage. Due to higher voltages, the 2.12 ${\upmu }$m particles can travel larger distances away from the electrode array, reach regions with higher fluid velocity (parabolic flow profile) and can consequently cover more distance per frequency cycle. This results in a faster simulated elution. The significantly lower predicted $R_{\mathrm{s}}$ compared to the experiments is a combination due to diverging $T_{\mathrm{max}}$ of both particles in comparison to the experiments and broader peaks (higher FWHM, Supplementary Fig. 4) in the simulation. Further, at $f_{\mathrm{c}} = {280}~\text {kHz}$, the simulation predicts significantly higher resolutions compared to the experimentally determined $R_{\mathrm{s}}$. $T_{\mathrm{max}}$ for both particle types match quite well across all voltages, leaving the width of the residence time distribution as diverging parameter (Eq. 4). Generally, a high resolution is achieved by a large time between the maxima of two peaks in combination with a small peak width. When the resident times show only small differences (small $\Delta T_{\mathrm{max}}$), the resolution becomes sensitive to small differences of the width of the residence time distributions (FWHM) when comparing experiment and simulation. Consequently, the reason for the disagreement concerning the $R_{\mathrm{s}}$ between experiment and simulation is the stronger peak broadening in the experiments and minor differences in $T_{\mathrm{max}}$. As soon as the 3.1 ${\upmu }$m particles experience retardation due to their nDEP dominated behaviour, their peaks begin to broaden (Supplementary Figs. 3 and 4). This peak broadening of the 3.1 ${\upmu }$m particles can also be seen in the elution profiles (Fig. 3, bottom row, middle and right panel, solid green line). In the simulation the particles behaviour is not as inhomogeneous as in the experiments, resulting in narrower peaks. When the larger particles are showing predominantly negative dielectrophoresis, they migrate close to the ceiling of the channel. The electric field is here much lower compared to the bottom, which is the location of the electrode array. In combination with the lower dielectrophoretic force ($\mathbf{F} _{\mathrm{DEP}}\propto \nabla |\mathbf{E} |^2$) this leads to inhomogeneous retention times, as now other effects such as unspecific particle-wall interactions or other fluid movements (e.g. electrothermal, buoyancy, AC electro-osmosis, hydrodynamic lift) can influence the particle movement. Additionally, as the DEP force decreases, particles travel shorter distances orthogonally to the wall due to DEP and consequently, cover less distance per frequency cycle. This could be compensated by adjusting the distribution with which the particles are released from the wall (see “Simulation model” section). Without detailed insight into the reasons behind these interactions, however, this seems like an arbitrary fit within the model. For achieving rapid separation with high resolution, consequently, pDEP dominated behaviour seems favourable according to the experiments.

Overall, the simulation gives valuable insight into the particle behaviour and trajectories in the channel and is able to support the process design. Additionally, it can be used to study the impact of side effects, since the simulation is able to isolate the movement due to drag, gravitation and dielectrophoresis and therefore a significant divergence between experiment and simulation suggests the presence of side effects.

### Material-selective separation

In this section, we will demonstrate the separation of polystyrene particles of almost equal size based on their surface functionalization. Firstly, we determine the crossover frequency using fixed-frequency experiments. Then, we input the crossover frequency into the simulation to find ideal separation parameters. Finally, we will use these parameters to separate the particles efficiently in an experiment. The separation was conducted using the already characterized 2.12 ${\upmu }$m PS particles without surface functionalization (plain) and 2 ${\upmu }$m carboxy functionalized PS particles (COOH).

The fixed-frequency experiments (see Supplementary Fig. 2) suggest a crossover close to 210 kHz for the carboxylated particles, resulting in a surface conductance of $K_{\mathrm{s}}=$ 0.6 nS, significantly lower compared to $K_{\mathrm{s}}$ of the plain particles ($K_{\mathrm{s}}=$ 0.9 nS). The voltage for separating theses two particle types was fixed at 160V$_{\mathrm{pp}}$ because this voltage showed the highest separation efficiency before. Modulation frequency (300 mHz) and bandwidth (240 kHz) remain unchanged. Since no training data for the carboxy modified particles was available before the experiments, they were simulated with the experimentally determined surface conductance ($K_{\mathrm{s}}=$ 0.6 nS). Compared to the plain 2.12 ${\upmu }$m particles, the surface conductance is lower. Therefore, the carboxy particles are expected to show balanced pDEP and nDEP movement or, with increasing center frequency, an nDEP dominated behaviour similar to the 3.1 ${\upmu }$m plain particles at center frequencies between 200 kHz and 300 kHz. Although the crossover frequency of the carboxy 2 ${\upmu }$m and plain 3.1 ${\upmu }$m particles are comparable, the mobility deviates significantly ($F_{\mathrm{DEP}}\propto d_{\mathrm{p}}^3$) leading to a more challenging separation task.

**Figure 4:**
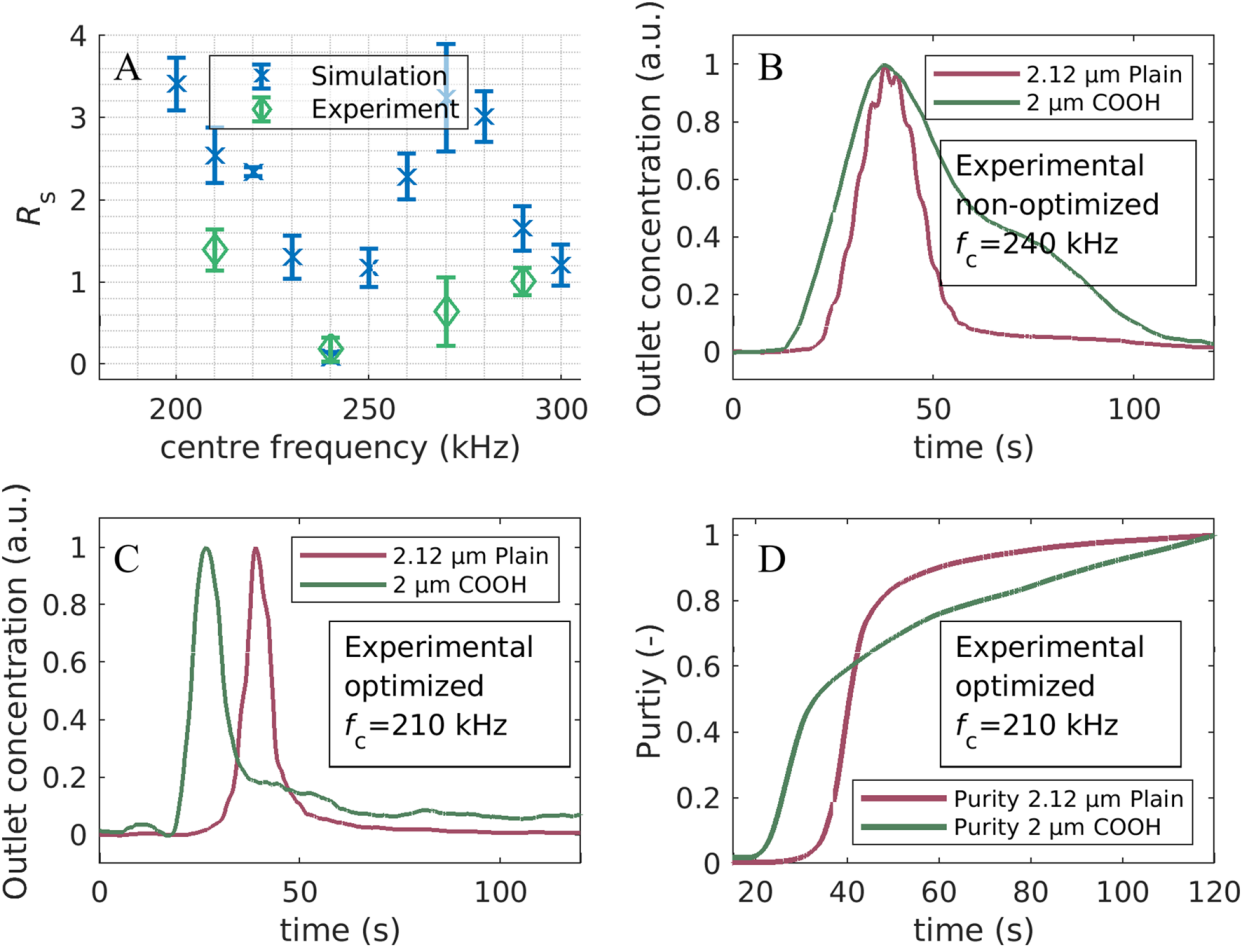
(**A**): Simulated (blue) and experimentally determined (green) resolution $R_{\mathrm{s}}$ at different center frequencies ($f_{\mathrm{c}}$). Simulations were repeated 5 times. (**B**): Chromatogram of a non-optimized separation of 2 ${\upmu }$m carboxy functionalized and 2.12 ${\upmu }$m plain PS particles at a $f_{\mathrm{c}}= {240}\;\text {kHz}$. (**C**) Chromatogram of an optimized separation of 2 ${\upmu }$m carboxy functionalized and 2.12 ${\upmu }$m plain PS particles at $f_{\mathrm{c}}= 210\,\text {kHz}$. (**D**) Purity as a function of time at $f_{\mathrm{c}}= 210\;\text {kHz}$.

The simulated resolutions (Fig. 4) show a first maximum at the lowest simulated center frequency. In this setting the carboxy particles show an almost balanced pDEP and nDEP movement and are therefore eluted first, while the better polarizable plain particles experience retardation due to pDEP. With increasing centre frequency the resolution reduces significantly as now both types of particles experience balanced pDEP/nDEP behaviour and thus, only small retardation. The minimum is reached at 240 kHz. Afterwards the resolution increases again, leading to a second peak at 270 kHz center frequency at which the plain particles experience a balanced pDEP/nDEP behaviour in contrast to the carboxylated particles which are now slowed down due to showing predominantly nDEP. At even higher center frequency, both particles show predominantly nDEP and the resolution is again low. Compared to the separation with respect to size (Fig. 3), the resolution is generally lower, which is expected because the magnitude of the DEP force depends less strongly on surface functionalization than on size.

Experiments then were conducted for four different sets of frequencies to test the separation in the experiment. The selected center frequencies were $210,\ 240,\ 270$ and 290 kHz. The best experimental separation resolution was achieved at a cetner frequency of 210 kHz with a value of $R_{\mathrm{s}}=1.39\pm 0.25$. This is lower compared to the simulation but the setting allows a chromatographic separation as predicted by the simulation (Fig. 4, bottom row). The simulation suggested a minimum of the separation efficiency at a centre frequency of 240 kHz. At this frequency an experimental separation was also not possible, resulting in a resolution of $R_{\mathrm{s}}=0.18\pm 0.15$ (Fig. 4, top right). To check whether with increasing frequency the resolution increases again, higher centre frequencies were tested. At a centre frequency of 270 kHz a resolution of $R_{\mathrm{s}}=0.64\pm 0.42$ was measured, whereas 290 kHz as the centre of the modulation spectrum resulted in an increase of the resolution up to $R_{\mathrm{s}}=1.02\pm 0.17$. Similar to the value at 210 kHz these values are below the simulative predicted ones, but the experiments do mirror the trends provided by the simulation.

In addition to the elution peaks, the purity (Eq. 5) is also plotted in Fig. 4. This provides another parameter besides retention time and resolution. Before a relevant amount of plain particles elute from the channel about 60 % of the carboxylated particles are crossing the measurement area. Furthermore, over 80% of the plain particles are eluted within a few seconds, which is crucial for a good separation.

## Conclusion

To conclude, we have used simulation and experiments to demonstrate three different particle behaviors in frequency-modulated chromatography, i.e., retardation due to nDEP or pDEP-dominated behavior or a balanced behavior leading to no retardation. We have firstly addressed size-selective separation of two different PS particles to investigate the particle retention mechanisms. Here, the simulation model supported our previous hypothesis. We then addressed the more challenging material-selective separation of particles of equal size to show the power of the simulation method: We used the simulation to find suitable operating parameters which allow a separation of two equally sized 2 ${\upmu }$m PS particles with different surface functionalization.

In the future, our simulation model can be used as a valuable tool to design operating schemes capable of addressing more complex separation tasks, for example shape sensitivity or heterogeneous samples, or to study how a reduction of the applied voltage would be possible for handling sensitive samples such as cells. To address biological particles we have to reduce the applied voltage while maintaining the ability to perform a chromatographic separation. This can be possible, for example, via geometrical optimization. Since biological systems typically have higher medium conductivities than used here, significant heat could develop if the voltage is not reduced. Additionally, high electric field strength could lead to irreversible electroporation of the suspended cells. The simulation does not always match the experiments exactly, which could only be achieved using extensive fitting considering the complex trap and release cycles. Nevertheless, the simulation can be used to perform design optimizations or to perform extensive parametric studies without the requirement to invest time and money on equipment and particles.

## Methods

The principle behind the fixed-frequency and frequency-modulated experiments were presented in the “Introduction” section for readability of the manuscript. However, experimental and simulative details are presented in the following.

### Functionality of frequency-modulated DPC

The suspended particles are injected into the channel and transported further by a carrier flow. During the experiments the particles are carried over an electrode array (Fig. 1A,B) and consequently subjected to an electric field. In this method, to generate trap and release or deceleration and acceleration cycles, the frequency of the electric field is not kept constant but modulated. In contrast to techniques published before, to the best of the authors knowledge, in this technique a modulation spectrum is chosen that generates pDEP and nDEP for all suspended particles during short periodic cycles rather than trapping one species first and releasing it after a different species was eluted from the channel. Therefore, the method does not dependent on strongly diverging polarizability of the particle mixtures for separation. Instead, it can be used to resolve minute or even overlapping distributions of the polarizability of particles. Using this approach a fast chromatographic separation can be achieved^33^.

Modulating a sinusoidal voltage by a triangular function results in periodic changes of the frequency between two values (Fig. 1E). The centre of this frequency range is called centre frequency ($f_{\mathrm{c}}$). During the modulation, particles may show pDEP in one part of the frequency range and nDEP in another one. Consequently, three different particle behaviours can be distinguished, as long as the crossover frequency is between the maximum and the minimum value of the modulation spectrum. Firstly, when particle shows more pDEP than nDEP in the applied frequency range, they tend to migrate towards the field maxima, which are located at the bottom of the channel at the electrode edges. Since in the channel a parabolic velocity profile is present due to the low Reynolds number, particles close to the wall are significantly slowed down either by low fluid velocity or by trapping. Consequently, their residence time in the channel is increased. Secondly, when a particle shows a balanced pDEP/nDEP response, the particles spend less time in low velocity regions due to the constant movement orthogonal to the fluid flow and therefore are only retarded minimal. Finally, when a particle predominately shows nDEP it migrates towards the ceiling of the channel and is slowed down there. Since the electric field gradients are smaller at the ceiling, trapping becomes more unlikely and migration velocities due to DEP are lower.

### Determination of crossover frequency

As presented above, the direction dielectrophoretic movement is, among other things, influenced by the particles conductivity and its size. However, the conductivity of the particles is unknown and needs to be evaluated prior to the DPC experiments or simulations. In this work, model PS particles are used. Since their material conductivity is negligible, the surface conductance has a significant impact on the polarization (Eq. 3). At suitable medium conductivities, PS particles are know to show positive dielectrophoresis at low frequencies due to the surface conductance and negative dielectrophoresis at high frequencies since the permittivity is much smaller compared to the surrounding medium^17,23^.

In the literature, multiple ways are presented to determine the dielectric properties and consequently the crossover frequency or vice versa. For example, the crossover can be measured by observing the particle movement when subjected to various frequencies^23,36^ or using electrorotation^34^. Even commercial and label-free systems are available by now which provide a rapid analysis of the frequency response of (biological) particles^37^.

An approach compatible with the DPC set-up was proposed by Sano et al.^29^. They stated that in dielectrophoretic particle chromatography a particle passes through a channel when the polarisation is negligible. This is valid for particles that are subjected to an electric field with a frequency which is close to the crossover frequency of the suspended particle and other effects such as hydrodynamic lift and gravitation are negligible. Gascoyne and coworkers ^19,30^ used a similar approach in batch-mode DEP field-flow fractionation and made it applicable for deformable particles.

By testing differed frequencies subsequently, the crossover frequency can be approximated by comparing the elution profiles of fixed-frequency DPC experiments to elution peaks where no voltage is applied (Fig. 1C).

### Experimental details

The microfluidic device has been described in detail in a previous publication^33^. Briefly, the $h=$ 80 ${\upmu }$m high microfluidic channel is made from PDMS, has a width of 2 mm and a length of about 17 cm. The channel is bonded to an electrode array using PDMS as a thin intermediate layer, which also is meant to reduce particle adhesion to the electrodes. The electrodes have a width and a spacing of 100 ${\upmu }$m and are connected to a single channel amplifier (A400, Pendulum Instruments, Sweden) which provides a constant amplification factor over a large bandwidth. The signal is generated by a signal generator (Rigol DG4062, Rigol Technologies EU GmbH, Puchheim, Germany) and controlled using a digital oscilloscope (Rigol DS2072A, Rigol Technologies EU GmbH, Puchheim, Germany). The particles were observed at the outlet using a Nikon TS2R-FL inverted fluorescence microscope (Nikon Instruments Europe BV, Amsterdam, The Netherlands), a white light source (XCite 120 PC, Excelitas Technologies Corp., USA), a triple-bandpass (DAPI/FITC/TRITC) and a USB RGB camera (GS3-U3-51S5C-C, FLIR Systems Inc., USA). Resident time distributions were obtained by segmenting and processing the video files from the experiments with MATLAB.

The particles were purchased from Polysciences, Inc. (USA) and suspended prior to the experiments in the medium. The suspension in all experiments has a conductivity of 2 ${\upmu }$S cm$^{-1}$. To produce the medium per 100 ml pure water (OmniaTap 6 UV/UF, stakpure GmbH, Germany), we add 2 ml of 1 % Tween 20 and 3μL of 0.01 M KOH to adjust the pH value. Further, KCl was added to adjust the conductivity to a value of 2 ${\upmu }$S cm$^{-1}$. The volume flow in all experiments was 5 mL h$^{-1}$ and the injection was conducted at $t= {10}~{\text s}$ by opening a manual 4 way valve (H&S V-101D, IDEX Health & Science, USA) for two seconds. The flow was generated by two syringe pumps (Legato 200 & 270, KD Scientific Inc., USA).

To quantify the outcome of the separation we use the resolution $R_{\mathrm{s}}$, which can be defined as

4 $$\begin{aligned} R_{\mathrm{s}}=\frac{\Delta T_{\mathrm{max}}}{\frac{1}{2}(w_1+w_2)}, \end{aligned}$$

with $T_{\mathrm{max}}$ being the maximum of the residence time distributions and $w_{\mathrm{x}}$ the full width at half maximum (FWHM). In addition to the resolution the purity of each fraction can be used to describe the outcome of an separation, which here is defined as

5 $$\begin{aligned} \frac{\sum _0^t I_{x}(t)}{\sum _0^{t= {120}{\text{s}}}I_{x}(t)}, \end{aligned}$$

by using *t* as time and $I_{x}(t)$ as fluorescence intensity at time *t*. This sum is normalized by its maximum cumulated intensity and therefore always reaches 1 at the end of the experiment ($t= {120}~{\text s}$).

### Simulation model

**Figure 5:**
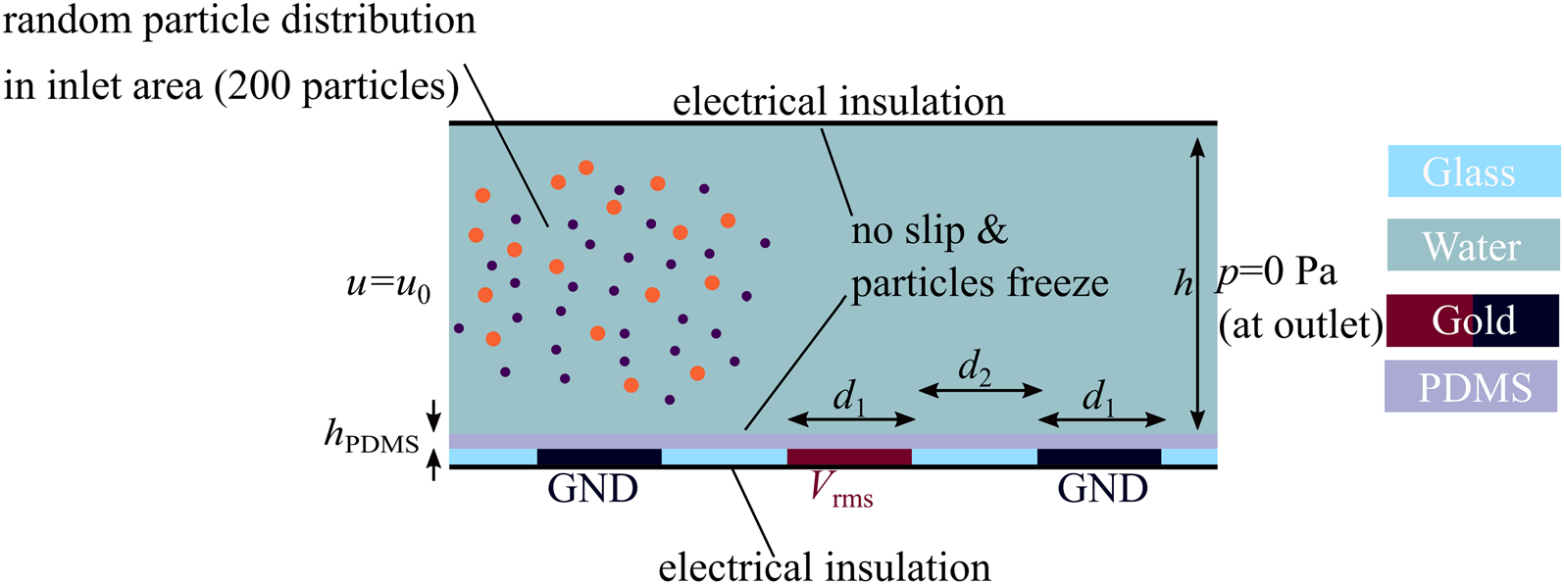
Important boundary conditions and materials of the simulative model. Important parameters: height of the channel $h=$ 80 ${\upmu }$m, width $d_1$ and spacing $d_2$ of the electrodes is 100 ${\upmu }$m. Inlet velocity 8.68 mm s$^{-1}$ and insulation thickness $h_{\mathrm{PDMS}}=$ 1.75 ${\upmu }$m. At the inlet 200 particles were randomly distributed in a 1.5 cm $\times$ 60 ${\upmu }$m area being 10 ${\upmu }$m away from bottom and ceiling.

To investigate the particle movement and to isolate effects, we build a simulation model using COMSOL Multiphysics linked to MATLAB. Boundary conditions are necessary, which were selected in accordance with the literature^18,38^ and are shown in Fig. 5. More details of the simulation model as well as a mesh independence study can be found in the supplement.

To compare experimental and simulative retention times of the particles quantitatively, a two-dimensional full scale model was chosen as basis for the simulation. Within the model three different sections, the static electric and velocity fields, particle tracing, and the MATLAB-COMSOL interaction, can be distinguished. The laminar flow ($Re\ll 1$) profile is calculated using the Stoke’s approximation for low Reynolds numbers. The inlet velocity can be obtained by dividing the volume flow by the area of the microchannel. As outlet boundary condition a constant pressure (0 Pa) is used. In combination with a no slip condition at ceiling and bottom a parabolic flow profile is calculated.

The electric field in experiment and simulation is generated by an electrode array and simulated at the center frequency of the modulation spectrum. In these arrays an electrode with a applied potential of $V_{\mathrm{RMS}}$ is neighboured by two electrodes on GND (0 V) potential (Fig. 5). A thin PDMS layer is placed on top of the electrodes. The thickness has not been determined experimentally but is significantly below 3 ${\upmu }$m according to literature^39^. It has been used as a fitting factor and the best match between experiment and simulation was achieved when using $h_{\mathrm{PDMS}}=$ 1.75 ${\upmu }$m. Placing PDMS as dielectric material on top of the electrode array generates a high-pass filter. The effect was simulated and implemented into to the model (Supplementary Fig. 1). The medium was simulated with a conductivity of $\sigma _{\mathrm{m}}= {2}~{{\upmu }\mathrm {S} \;\text {cm}^{-1}}$ and a relative permittivity of 78 whereas the particles were simulated with a substatially lower relative permittivity of 2.7 and a conductivity calculated according to Eq. (3) using $\sigma _{\mathrm{bulk}} = 0$. Coupling of fluid field and electric field was not added, since the experiments were conducted at low medium conductivity and sufficiently high frequencies. The first point does reduce the effect of electrothermal movement (heat loss density $=\sigma _\text m \mathbf{E} ^2$) whereas the latter suppresses the influence of AC electroosmosis^40,41^.

However, in microfluidics unspecific adhesion, (electro-)thermal flow, hydrodynamic lift, particle-particle interactions and/or electrokinetic phenomena can play an important role, but are hard to quantify and therefore to implement. As a result, experimental training data was used to get a good match by adjusting some parameters of the simulation in a reasonable range. The adjusted parameters were PDMS isolation thickness and the surface conductance (“Size-selective separation” section) as well as the particle release offset (see below).

The second part of the simulation is the particle movement description. Particles are experiencing positive and negative dielectrophoresis, viscous drag, and gravitation. All particles are assumed to be massless, which is reasonable given their small stopping distance, to reduce the computational effort. Additionally, as soon as particles reach the ceiling or bottom they are assumed to be trapped, which is not always true in reality. Once particles are trapped in the simulation they stay at their location. This is not valid for a DPC experiment, which leads to the third part of the model which is formed by the COMSOL-MATLAB interaction. The ${\mathrm {Re}}(CM)$ needs to be calculated for each time step to implement the impact of the frequency modulation into the COMSOL model and consequently produce pDEP and nDEP movement of the particles with respect to their properties. To implement the frequency changes into the simulation a sawtooth function was used in COMSOL which then was used as feed for a calculation of the Clausius-Mossotti factor as a function of time/frequency. This procedure allows to reduce the computational cost of the simulation because is not needed to calculate the electric field in every single time step of the time dependent solver.

Using MATLAB, the movement of the particles through the channel is divided into multiple parts. In the experiment the valve to inject the particles is opened for two seconds. In this time period, particles are entering the channel at different heights (*y*-positions) and times. Due to the constant flow they are at different (*x*-)positions along the channel. Consequently to reproduce this kind of peak in the simulation, particles are initialized in an area rather than on one point or line. For this purpose we added an inlet area of 1.5 cm in front if the simulated channel where no electrodes are existent and $n=200$ particles per type are randomly placed at the beginning in a range of heights between 10 and 70 ${\upmu }$m.

After the particles are released they experience dielectrophoresis and may eventually reach a boundary where they freeze. Consequently, at sufficient high voltages no particles would exit the channel in the simulation. To overcome this issue, a MATLAB script checks ${\mathrm {Re}}(CM)$ for changes of its sign and stops the simulation as the value reaches zero. At this point the model checks for particles adhering to the wall and repositions them up to 10 ${\upmu }$m orthogonal to the wall into the channel. The extend of the manipulation of the particles position is randomly chosen between 0 and 10 ${\upmu }$m to incorporate the inhomogeneous nature of particle-wall interactions, which effectively can lead to broader, less pronounced elution peaks. Particle positions are logged to calculate residence time distributions. Since the model contains random components multiple runs are necessary to check for statistical validity (Supplementary Fig. 9).

## Supplementary information

Supplementary Information.

## Data Availability

The datasets generated and/or analyzed during the current study are available from the corresponding author on reasonable request.
