# Supplementary material for: Separating microparticles by material and size using dielectrophoretic chromatography with frequency modulation
Source: Sci Rep. 2021 Aug 19;11:16861. doi: 10.1038/s41598-021-95404-w (PMC8376941; doi:10.1038/s41598-021-95404-w)
Supplement: Supplementary file 1 — Supplementary Information. [file 41598_2021_95404_MOESM1_ESM.pdf]

Supporting information: Separating microparticles by material and size using dielectrophoretic chromatography with frequency modulation

Jasper Giesler[1], Laura Weirauch[1], Jorg Thöming[1,2], Michael Baune[1], and Georg R. Pesch[1,2,*]

[1]Chemical Process Engineering, Faculty of Production Engineering, University of Bremen, Leobener Straße 6, 28359 Bremen,Germany
[2]MAPEX Center for Materials and Processes, University of Bremen, 28359 Bremen, Germany
[*]gpesch@uni-bremen.de

ABSTRACT

Separation of (biological) particles ($\ll 10\,\mu m$) according to size or other properties is an ongoing challenge in a variety of technical relevant fields. Dielectrophoresis is one method to separate particles according to a diversity of properties, and within the last decades a pool of dielectrophoretic separation techniques has been developed. However, many of them either suffer selectivity or throughput. We use simulation and experiments to investigate retention mechanisms in a novel DEP scheme, namely, frequency-modulated DEP. Results from experiments and simulation show a good agreement for the separation of binary PS particles mixtures with respect to size and more importantly, for the challenging task of separating equally sized microparticles according to surface functionalization alone. The separation with respect to size was performed using $2\,\mu m$ and $3\,\mu m$ sized particles, whereas the separation due to surface functionalization was conducted with $2\,\mu m$ particles solely. The results from this study can be used to solve challenging separation tasks, for example for particles with distributed properties and surface characteristics.

1 Experimental Set up

An image from a digital microscope of a section of the device is given in figure in 1. The videos for the calculation of the residence time distributions were recorded in the measurement area. This area is close to the outlet and right behind the last pairs of electrodes. Since an inverted microscope was used, videos were recorded from below the channel filming through the electrode chip. After the recording the video was compressed using ffmpeg and segmented with Matlab.

On top of the electrodes the thin PDMS layer produces a high-pass filter effect which can be simulated in COMSOL. For various thicknesses (figure 1) this effect could observed in the simulation. However, since the lowest frequency in the manuscript was 90 kHz no significant influence is to be expected in the experiments. The effect was implemented by multiplying the electric field with a frequency-dependent fit function so that $E = g(f) \cdot E_0$. $g(f)$ is determined through $g(f) = 1 - \exp^{-8.013e-5 \cdot f}$, with f the electric field frequency.

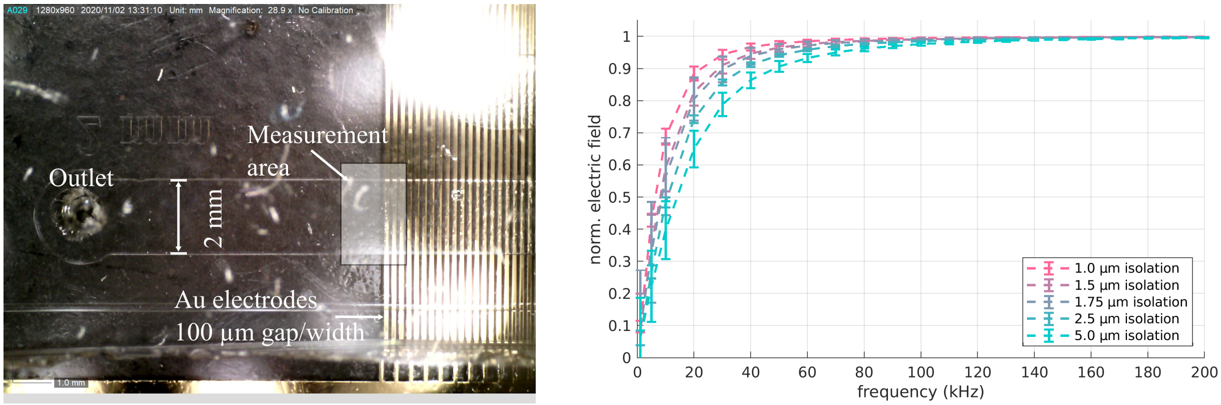

Figure 1. Left: Photograph (digital microscope) of the channel with important parameters of the set-up. Right: Normalized electric field strength of mesh points for various isolation thicknesses of a representative area (400 µm x 80 µm). The intensity of a point is normalized to the value of that point at 300 kHz. The standard deviation provides information about how different the single points are affected by the high-pass filter effect.

2 Results

2.1 Fixed-frequency data 2 μm

For the plain 2.12 μm and the carboxylated 2 μm particles results of the fixed-frequency measurements are displayed in figure 2. The best match between the experiments with no applied voltage and an voltage at fixed frequency was found at 290 kHz resulting in an surface conductance of $K_s = 0.9\,\mathrm{nS}$. In contrast to the plain particles, the carboxylated 2 μm spheres show a much lower crossover frequency (figure 2). This results in a measured crossover of $K_s = 0.6\,\mathrm{nS}$. Without applied voltage the particles elute at 27.5 s.

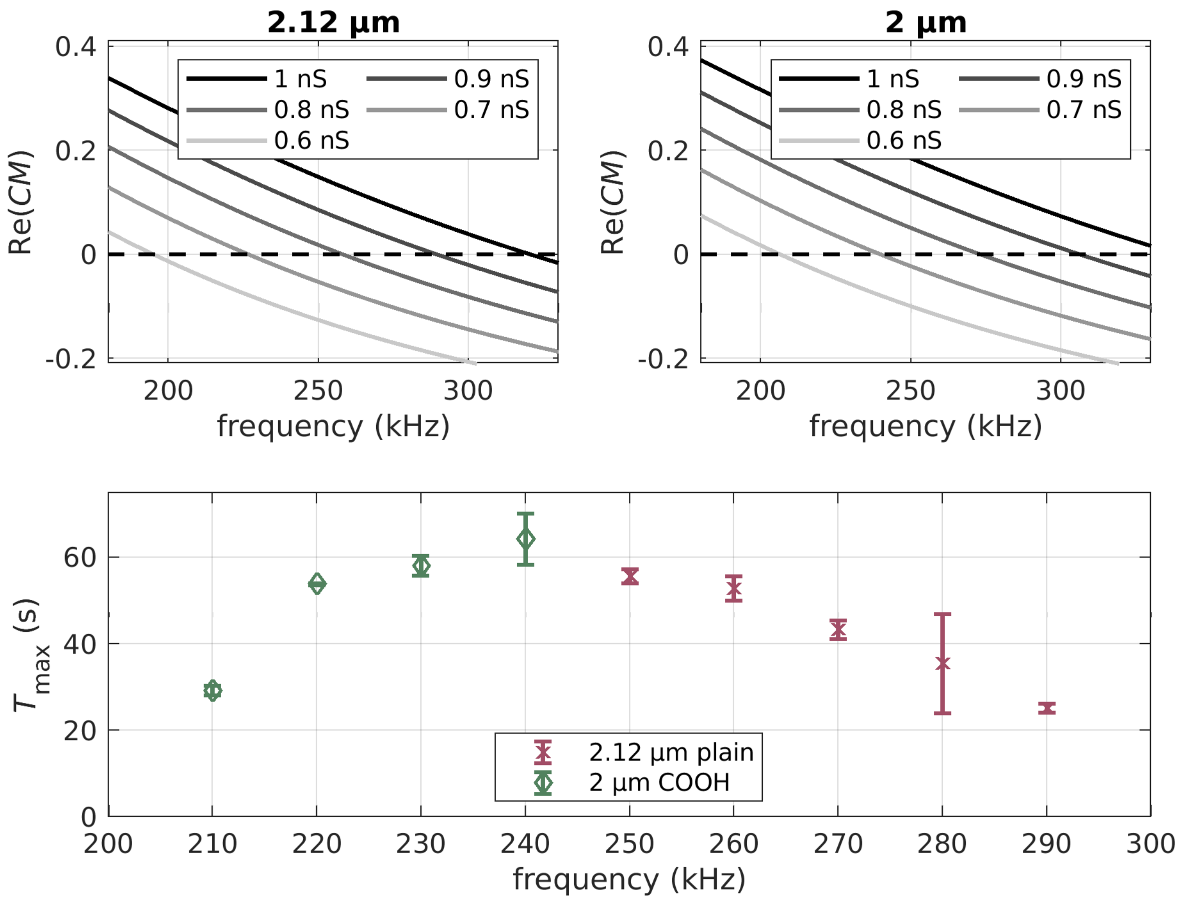

Figure 2. Top: Calculated values of the real part of the Clausius-Mossotti equation for different surface conductances. $d_p = 2\,\mu\mathrm{m}$ PS particles with a carboxy functionalization and $d_p = 2.12\,\mu\mathrm{m}$ PS particles without functionalization in a $2\,\mu\mathrm{S\,cm^{-1}}$ suspension. Bottom: Maximum of the residence time distributions for different frequencies.

2.2 FWHM data

The maximum of the peaks matches well as shown in the main document. As described before, the simulated peaks of the 3.1 µm particles are more narrow than they are in the experiment, as soon as the particles experience a retardation due to nDEP dominated movement. This can be seen in figure 3. However, when the nDEP/pDEP ratio is balanced, experiment and simulation show a good agreement. Figure 4, which displays the full width at half maximum (FWHM) values for the 2.12 µm particles, shows a better match for experiment and simulation. Except for one case ($f_c = 210\,\text{kHz}$ and $V_{pp} = 80\,\text{V}_{pp}$), where in the simulation significant peak broadening becomes visible (large FWHM). In this case the small 2.12 µm particles do not show sufficient particle movement away from the electrodes in the simulation when they experience nDEP due to the low applied voltage and centre frequency. In reality in contrast, the particles are able to elute from the channel faster. This might be due to the freeze boundary condition in the simulation which seems not to be always valid for the particles. However, in general the FWHM values of the smaller particles are much lower than of the 3.1 µm particles. We assume, that this is because of the strong DEP close to the electrodes which might suppress other effects and leads to a movement dominated by dielectrophoresis.

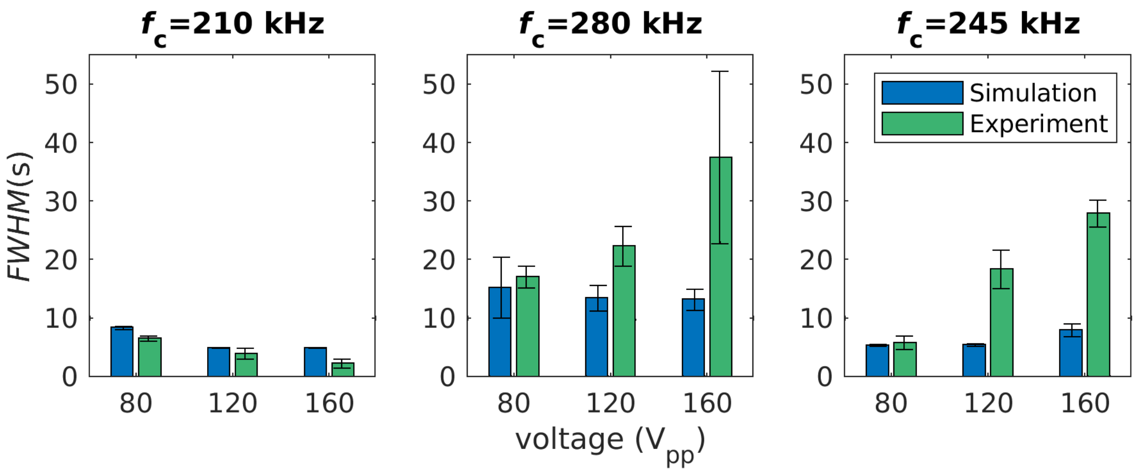

Figure 3. Full width at half maximum for the plain 3.1 µm at different centre frequencies and voltages. Both, simulation and experiments were conducted 5 times to check for statistical validity.

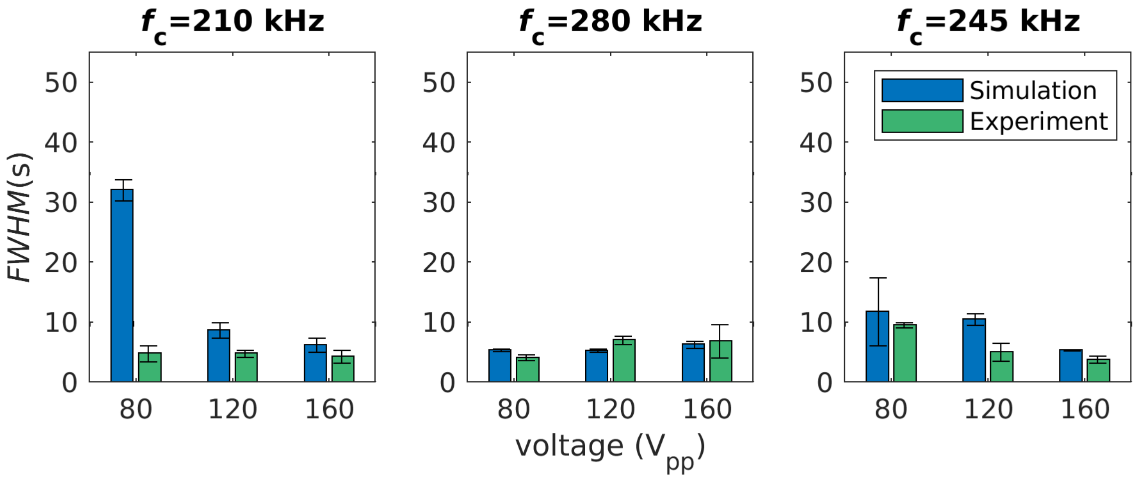

Figure 4. Full width at half maximum for the plain 2.12 µm at different centre frequencies and voltages. Both, simulation and experiments were conducted 5 times to check for statistical validity.

2.3 Size selective separation of 3.1 µm and 6.14 µm

To further investigate the nature of the offset of the surface conductance, experiments with larger diameter than 3.1 µm were conducted. The intention behind these experiments is to check whether the offset of the surface conductance, which is needed to harmonize experiment and simulation, vanishes for larger particles.

First, the crossover frequency and consequently the surface conductance was determined by conducting experiments at a fixed frequency (figure 5). The crossover of the 6.14 µm particles is in the range of about 90 kHz to 110 kHz resulting in a surface conductance of about 1 nS. When looking at the residence time distributions of 90 kHz, 100 kHz and 110 kHz two peaks are observable. The first peaks match with the maximum of the experiments without applied voltage but the second one is substantially later. This might be linked to a distribution of surface conductance of the particles. However, for lower frequencies than 90 kHz the particles showed significant adhesion to the channel and retardation. This can be due to pDEP and trapping at the electrodes. At frequencies above 110 kHz the particles are retarded as well which indicates the presence of substantial nDEP.

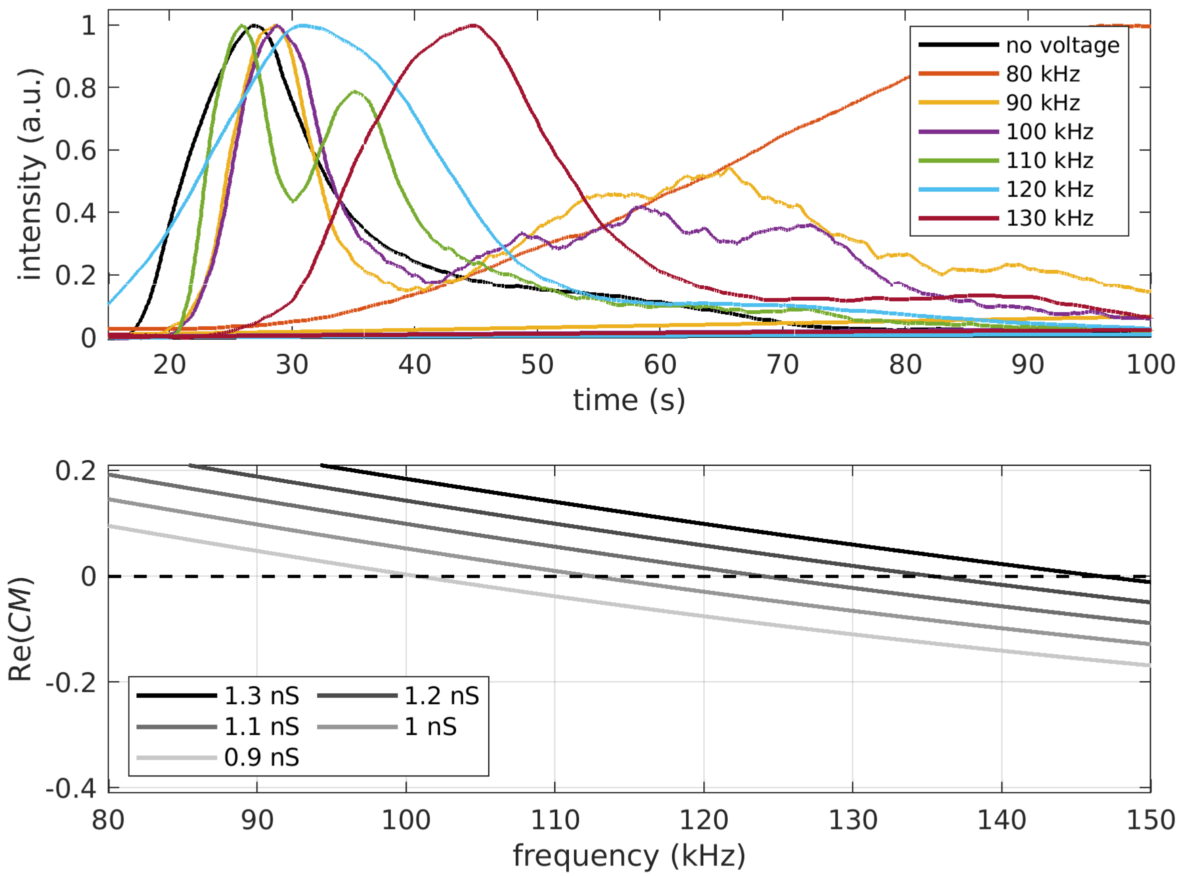

Figure 5. Top: Residence time distributions of 6.14 µm PS particles at 80 V_{pp} at various frequencies. Bottom: Calculated values of the real part of the Clausius-Mossotti equation for different surface conductances. $d_p = 6.14$ µm PS particles in a 2 µS cm^{-1} suspension.

As a second step the separation of 6.14 µm and 3.1 µm was conducted experimentally. Since the crossover frequencies are about 100 kHz apart and the size diverges significantly, the separation should be possible and was also show before[1]. Since the 3.1 µm do not show significant retardation at a centre frequency of 210 kHz (s. main document), this centre frequency was selected to separate the two particle sizes. The experiments were conducted at three different voltages and show a clear separation at these process parameters (figure 6 and table 1). Afterwards the experiment was simulated to check at what surface conductance the results of experiment and simulation show a good agreement. To achieve a agreement the simulated surface conductance of the 6.14 µm particles was needed to be raised to 1.3 nS. In figure 7 the correlation between surface conductance and crossover frequency is displayed for various particle sizes. This diagram shows that different diameters result in different

slopes regarding the interplay of crossover frequency and surface conductance. For larger particles the influence of the surface conductance on the crossover frequency is lower. Consequently, the correction needed to match experiment and simulation is comparable, in terms of change of the crossover frequency, to the correction of the 2.12 µm particles.

	80 V_{pp}	120 V_{pp}	160 V_{pp}
simulation	3.52 ± 0.38	3.7 ± 0.38	3.89 ± 0.36
experiment	2.79 ± 0.46	4.37 ± 0.51	4.36 ± 0.61

Table 1. Simulated and experimentally determined resolution of the separation of 3.1 µm and 6.14 µm PS particles at a centre frequency of 210 kHz.

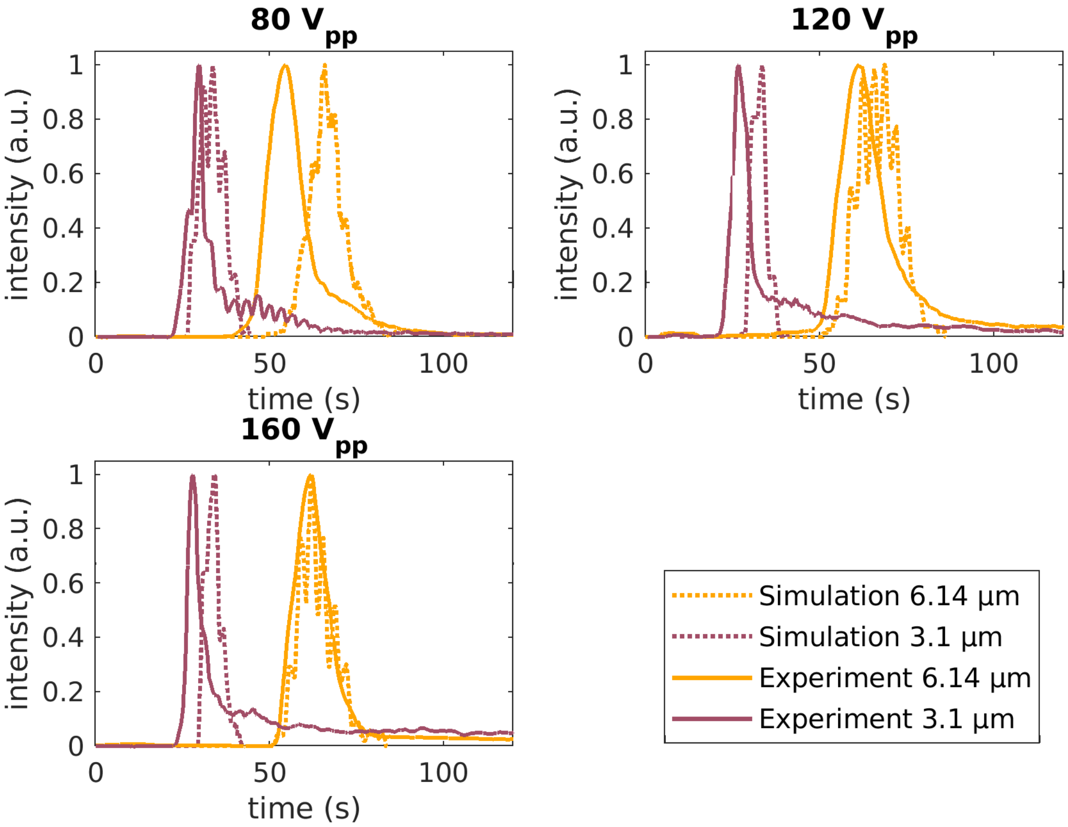

Figure 6. Overlay of separation simulated and experimentally conducted of 3.1 µm and 6.14 µm plain PS particles for three different voltages at a centre frequency of 210 kHz. Simulated surface conductance K_s for 3.1 µm particles was 1 nS and 1.3 nS for 6.14 µm particles. Both, simulation and experiments were conducted 5 times to check for statistical validity.

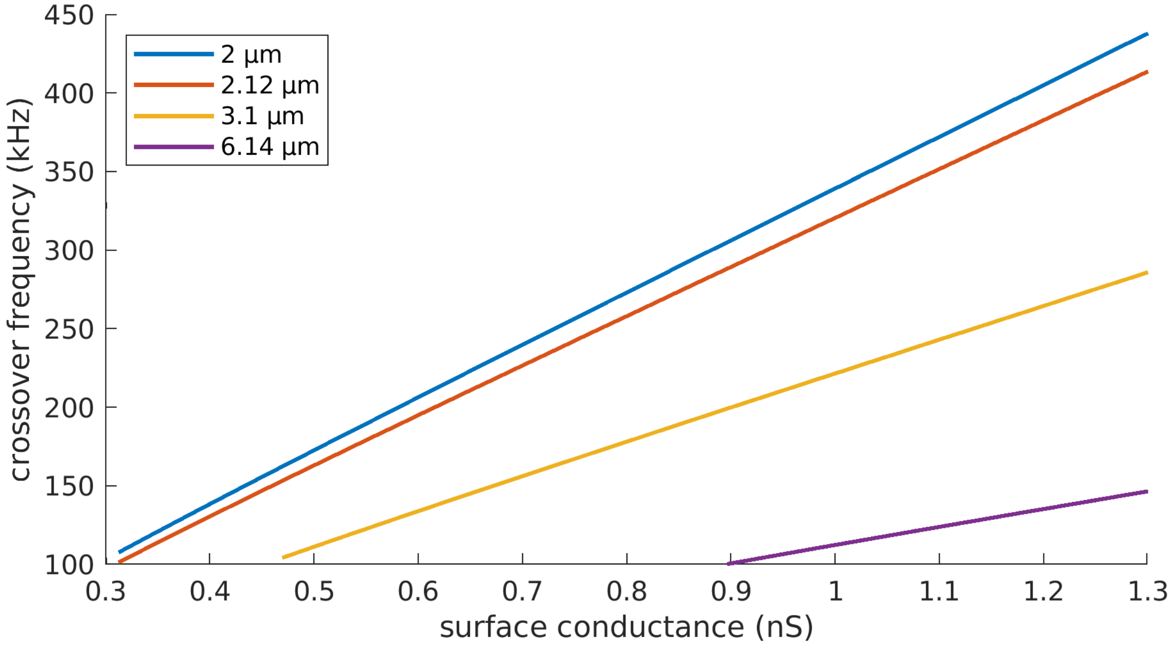

Figure 7. Dependency of the crossover frequency with respect to the surface conductance of PS particles with different diameters at a medium conductivity of $2\,\mu S\,cm^{-1}$.

3 Simulation

The simulations were conducted using Comsol Multiphysics 5.6 on a Xubuntu 20.04 system. The system features a AMD EPYC 7502P 32-Core CPU, 128GB DDR4-3200 RAM and a NVIDIA GeForce GTX 1660 (6 GB). The fluid flow was simulated using the creeping flow physics from the microfluidics module, constant pressure ($p = 0\,\mathrm{Pa}$) and a constant inlet velocity. At the bottom and the ceiling of the channel a no slip boundary condition was defined ($\mathbf{u} = 0$, with $\mathbf{u}$ as fluid velocity vector). The electric field is simulated using the build-in electric currents physics of the AC/DC module of COMSOL. The boundaries of the simulated system (inlet, outlet, ceiling of the channel and bottom of the electrodes) are defined as electrical isolation ($\mathbf{nJ} = 0$, with $\mathbf{J}$ the current density) and the electrodes with a prescribed voltage. Particles are simulated using the particle tracing module and are considered massless and only are affected by dielectrophoresis, viscous drag and gravitation. As soon as a particle reaches bottom or ceiling of the channel, a freeze boundary ($\mathbf{v} = 0$, with $\mathbf{v}$ the particle velocity vector) takes action and immobilizes the particle until its position is redefined using MATLAB. The connection of MATLAB and COMSOL was achieved by utilizing the LiveLink for MATLAB interface module developed by COMSOL. The governing equations are described in the literature in more detail[2–4].

3.1 Grid independence and solver settings

To check for mesh independent results four different meshes were tested. For comparing the different meshes a simulation was used in which particles were released at the inlet of the channel (uniformly distributed over the channel height) at $t = 0s$. These particles experienced drag, gravity and positive dielectrophoresis ($\mathrm{Re}(CM) = 1$). After simulating the trajectories, these were exported to MATLAB and the time until the particles reach the channels bottom are compared (figure 8). The solution was found to be mesh independent for the processed meshes and the mesh with the presetting *finer* was used in the simulation.

The default solver were used for both, the stationary (direct MUMPS solver) and the time dependent (iterative GMRES solver) study. The particles positions were stored every 0.05 s and exported to MATLAB.

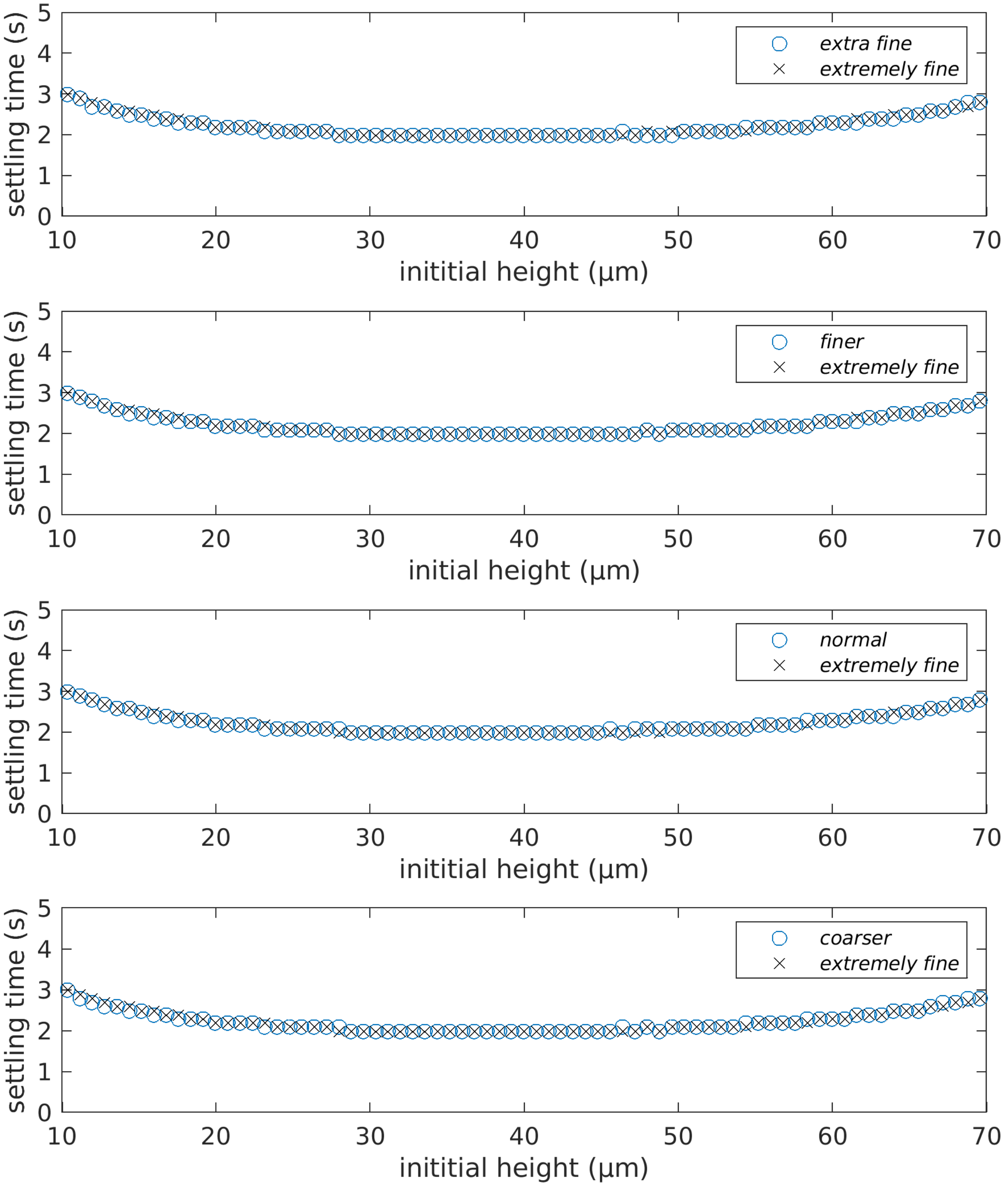

Figure 8. Study for determining the mesh independence for different meshing presetting. The number of elements (triangular + quadrangular elements) are 3.59×10^6 cells (extremely fine), 2.87×10^6 cells (extra fine), 2.58×10^6 cells (finer), 1.91×10^6 cells (normal) and 1.04×10^6cells (coarser).

3.2 Statistical analysis

The simulation contains mainly two random parameters. First, the 200 particles per species are randomly distributed in the inlet area in the simulation. Second, the release offset the particles get, as soon as the crossover frequency is reached during the simulation. We conducted 100 simulations with the very same particles and selected a case witch showed a distribution of resolutions. The selected case was the material selective separation with a centre frequency of 210 kHz at 160 V_{pp}. The distributions of resolution, of the maximum of the peaks and the full width at half maximum (FWHM) are shown in figure 9. The position of the maximum of the peaks only varies slightly, whereas the FWHM values of the 2.12 µm show a variation. Because of their longer residence time in the channel more distributed elution times become more likely due to the stochastic character of the simulation. This variation is the reason for the distribution of the resolution and shows the importance of conducting multiple simulations for the same parameter to generate statistical validity.

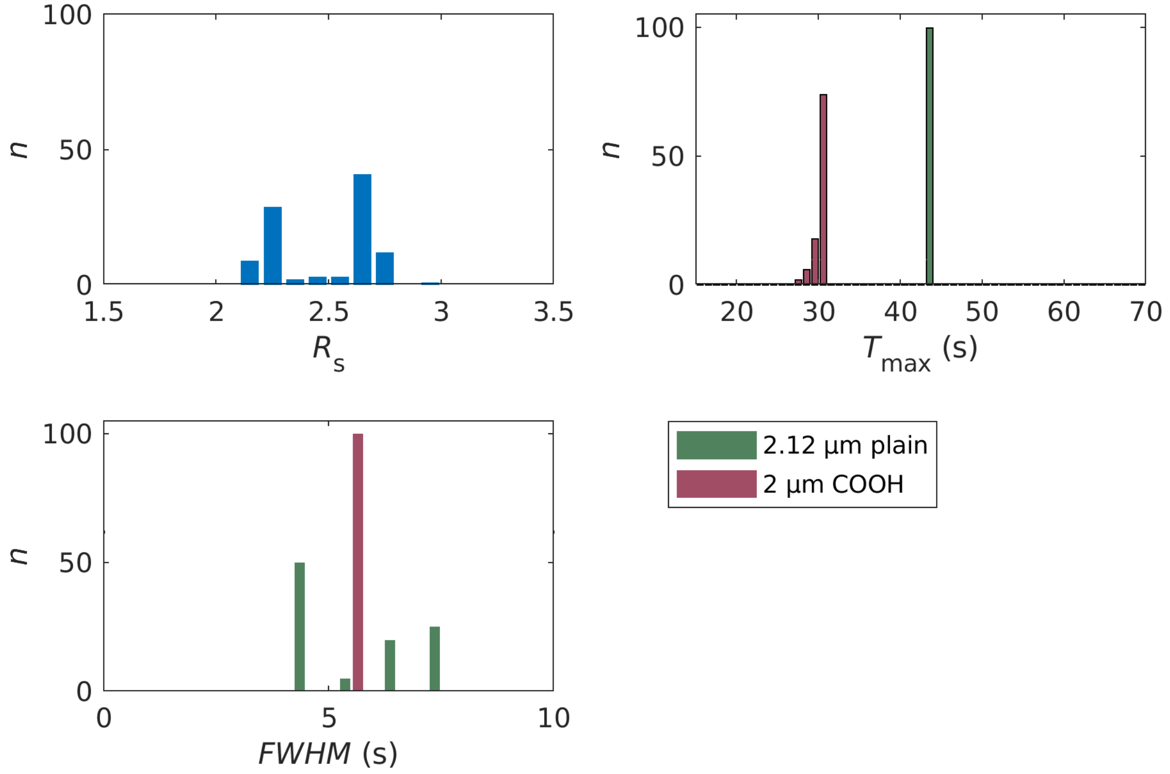

Figure 9. Statistical analysis of a separation of 2.12 µm plain particles and 2 µm carboxy particles at 160 V_{pp}, frequency window 90 kHz to 330 kHz (centre frequency 210 kHz) and a modulation frequency 300 mHz. Top left: bar plot of the resolution. Interval size is 0.1. Top right: distribution of the position of the maximum of the peaks. Bottom left: Full width at half maximum (FWHM) distribution of the simulation data. Interval size for T_{max} and $FWHM$ is 1 s.

References

1. Giesler, J. *et al.* Polarizability-dependent sorting of microparticles using continuous-flow dielectrophoretic chromatography with a frequency modulation method. *Micromachines* **11**, 1–14, DOI: 10.3390/mi11010038 (2020).

2. Pesch, G. R., Du, F., Baune, M. & Thöming, J. Influence of geometry and material of insulating posts on particle trapping using positive dielectrophoresis. *J. Chromatogr. A* **1483**, 127–137, DOI: 10.1016/j.chroma.2016.12.074 (2017).

3. Zhang, Y. & Chen, X. Dielectrophoretic microfluidic device for separation of red blood cells and platelets: a model-based study. *J. Braz. Soc. Mech. Sci. Eng.* **42**, 1–11, DOI: 10.1007/s40430-020-2169-x (2020).

4. Dalili, A. *et al.* Parametric study on the geometrical parameters of a lab-on-a-chip platform with tilted planar electrodes for continuous dielectrophoretic manipulation of microparticles. *Sci. Reports* **10**, 1–11, DOI: 10.1038/s41598-020-68699-4 (2020).
